# Mini-Review: The Contribution of Intermediate Phenotypes to GxE Effects on Disorders of Body Composition in the New OMICS Era

**DOI:** 10.3390/ijerph14091079

**Published:** 2017-09-17

**Authors:** Edna J. Nava-Gonzalez, Esther C. Gallegos-Cabriales, Irene Leal-Berumen, Raul A. Bastarrachea

**Affiliations:** 1 Facultad de Salud Pública y Nutrición (FASPyN), Universidad Autónoma de Nuevo León, Monterrey 64290, Mexico; ednajnava@hotmail.com; 2 Facultad de Enfermería, Universidad Autónoma de Nuevo León, Monterrey 64290, Mexico; esther.gallegosc@gmail.com; 3 Facultad de Medicina y Ciencias Biomédicas, Universidad Autónoma de Chihuahua, México 31110, Mexico; ilealb62@yahoo.com.mx; 4 Department of Genetics and Southwest National Primate Center, Texas Biomedical Research Institute, San Antonio, TX 78227, USA

**Keywords:** GxE interactions, intermediate phenotypes, OMICS, diabetes, obesity, osteoporosis, phenome, exposome

## Abstract

Studies of gene-environment (GxE) interactions describe how genetic and environmental factors influence the risk of developing disease. Intermediate (molecular or clinical) phenotypes (IPs) are traits or metabolic biomarkers that mediate the effects of gene-environment influences on risk behaviors. Functional systems genomics discovery offers mechanistic insights into how DNA variations affect IPs in order to detect genetic causality for a given disease. Disorders of body composition include obesity (OB), Type 2 diabetes (T2D), and osteoporosis (OSTP). These pathologies are examples of how a GxE interaction contributes to their development. IPs as surrogates for inherited genotypes play a key role in models of genetic and environmental interactions in health outcomes. Such predictive models may unravel relevant genomic and molecular pathways for preventive and therapeutic interventions for OB, T2D, and OSTP. Annotation strategies for genomes, in contrast to phenomes, are well advanced. They generally do not measure specific aspects of the environment. Therefore, the concepts of deep phenotyping and the exposome generate new avenues to exploit with high-resolution technologies for analyzing this sophisticated phenome. With the successful characterization of phenomes, exposomes, and genomes, environmental and genetic determinants of chronic diseases can be united with multi-OMICS studies that better examine GxE interactions.

## 1. Introduction

In the past, disorders of body composition (OSTP, OB, and T2D) were thought to be independent of one another. These disorders share several common distinctive features, as they all have a genetic basis that interacts with the environment. The least integrated in the concept of body composition disorders is osteoporosis, since the links that relate to diabetes and obesity have been extensively studied [1]. There is the central regulation of bone remodeling through the hypothalamus and sympathetic nervous system, a pathway that regulates the metabolic fate and distribution of adipose tissue [2]. On the other hand, fat tissue, via signaling through the hypothalamus, can regulate bone mass as a means of controlling energy use and modulating insulin secretion and sensitivity [3]. The metabolism of glucose and the physiological effects of insulin are significantly affected when there is a metabolic alteration of the communication between bone and adipose tissue [4]. Most patients with diabetes are obese and sedentary. Women with T2D have a higher proportion of fractures, mainly of the hip, than women without T2D [5]. It has also been documented that bone loss is much higher in patients with poor control of their glucose levels than in those with diabetes who have good metabolic control [6]. As we have learnt more about bone and fat, it has become more accurate to conclude that these complex diseases are connected through multiple hormonal, neuronal, and environmental pathways. The effects of GxE interactions on molecular biomarkers or intermediate phenotypes, such as those involved in fat tissue metabolism, the insulin-glucose axis, and bone mineral turnover, have been reported in recent literature [7].

The aim of this paper is to discuss the role of intermediate phenotypes (IPs) in the context of gene-by-environment interactions on clinical outcomes. Although a unified definition for intermediate phenotype, endophenotype, or biomarkers is intended, these terms are mainly used to designate a connection between the genome, the environment (exposome), risk behaviors, and health outcomes. These IPs are placed at the crossroad where GxE and disease risk intersect. Given the broad area of gene-environment interactions on various chronic diseases and risk factors such as obesity, lipids, diabetes, cardiovascular disease, cancer, multiple sclerosis, pancreatitis, schizophrenia, bipolar disorder, Parkinson’s disease, and longevity [8], the scope of this paper is presented as a mini-review, one that it is limited to the disorders of body composition. This paper also highlights the importance of integrating the exposome and phenome with the new trend of investigating the genome through a multi-OMICS approach to better characterize GxE interactions. 

## 2. Gene-Environment Interaction

A general definition of a gene-environment (GxE) interaction frequently found in the literature refers to the fact that the effects of genes on a disease often depend on the environment or that the effect of the environment depends on the genotype [9]. The term is used to indicate that an effect is due to a mixture of environmental factors (nurture) and genetic factors (nature) [10]. In OB, T2D, and OSTP, the circulating protein products (metabolic biomarkers) derived from the expression of a gene (referred to as biological risk phenotypes) are continuous (quantitative) traits that exert a profound influence on the genetic susceptibility to develop such pathological processes, coupled with a deep interaction with environmental factors [11]. The phrase gene-environment (GxE) interaction implies that the direction and magnitude of the clinical effect that a genetic variant has on the disease phenotype can vary as the environment changes. In other words, the genetic risk for disease is modifiable in an environment-specific manner. Furthermore, an individual can inherit a predisposition for a devastating disease, yet never develop the disease unless exposed to the appropriate environmental trigger(s) [12]. GxE interactions can be described by using several models which take into account the various ways in which genetic effects can be modified by environmental exposures, the number of levels of these exposures, and the model on which the genetic effects are based. The choice of study design, sample size, and genotyping technology influence the analysis and interpretation of the observed GxE interaction. Genetic predisposition can be inferred from family history, the phenotype (for example, skin color), or the direct analysis of a DNA sequence. Environmental and lifestyle factors are measured in epidemiological studies using self-reported information; this can be obtained by interviews or questionnaires, from records or direct measures in participants (for example, anthropometry), or from biomarker-based inferences on environmental exposures [13].

## 3. Determinants of Unhealthy Eating Habits and Physical Inactivity: Risk Behaviors and Disorders of Body Composition as Health Consequences

Risk behaviors are factors or conditions that predispose a person to become ill, resulting in negative consequences. The combination of a lack of activity and inappropriate eating habits has been classified as an important factor contributing to diseases such as OB, T2D, and OSTP, among others [14]. These disorders of body composition have reached record proportions, by compromising adipose tissue metabolism, the insulin-glucose axis, and bone physiology [15]. There is extensive literature on how eating behaviors, physical activity, and a sedentary life style significantly influence the development of these disorders of body composition. Genetics must be combined with an “obesogenic” environment conducive to gaining weight [16]. The evolution of our “obesogenic” environment has been both rapid and multifactorial. There has been a tremendous increase in the availability of food, especially high-fat and/or high-calorie food, at the same time that there has been a decrease in the amount of individual physical activity. An additional factor is the attraction to the fast food industry with the low cost of many items making this high-fat, calorie-dense diet available to just about everyone. At the same time, our diets have taken a turn for the worse, and the amount of physical activity in our lives has decreased. A number of studies have suggested that the increasing prevalence of obesity is, in fact, more strongly related to decreased energy expenditure than to increased energy consumption. It is clear that an active lifestyle decreases the risk of several chronic diseases and improves our overall quality of life [17].

Several research studies have shown that these disorders share common associations and interactions. Bone mineral density (BMD) is used as an indirect indicator of the risk of fracture and osteoporosis. Studies indicate that genetic factors account for 50–85% of the predisposition to decreased BMD. In women, weight is the strongest predictor of BMD [18]. Zerwekh et al. [19] documented the deleterious effects of sedentary risk behavior on BMD reduction. These investigators reported reductions in BMD of 1% to 4% in the lumbar spine, femoral neck, and major trochanter in healthy individuals of both sexes after 12 weeks of being bedridden. Zilikens et al. [20] examined the relationship between BMD and body mass index (BMI), adiponectin, and insulin in 2631 overweight participants. They established that there is a positive association between android adipose tissue distribution and BMD. The authors concluded that the association found is deleterious for bone mineral turnover and bone quality. Kim [21] studied 906 postmenopausal women, 60 to 79 years of age, of a normal nutritional status. After adjusting for age, smoking, alcohol consumption, total calcium intake, and total energy consumption, they found that waist circumference was negatively correlated with BMD, while body weight was positively related to BMD. The percentage of body fat and waist circumference was much higher in the fracture group than in that without fracture. The authors concluded that excessive body fat accumulation and increased waist circumference are associated with low BMD and components of the metabolic syndrome. These research examples studying the biological effects on the variation of BMD help us to understand the susceptibility of BMD to environmental factors, and also show that the inclusion of bone turnover traits with risk behaviors and glucose and adipose tissue metabolic phenotypes gives the study design a higher integrative scope [22]. 

## 4. Intermediate Phenotypes 

**Figure 1:**
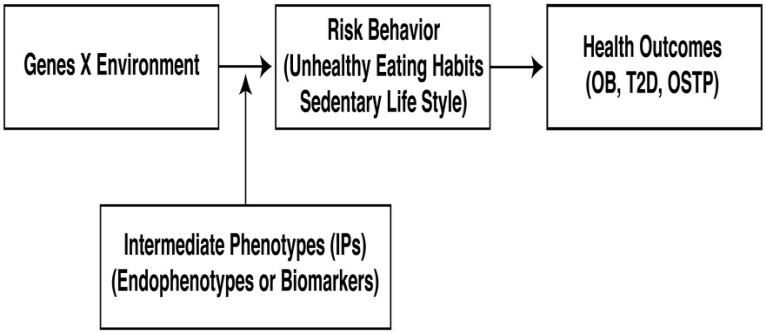
Basic predictive model showing how IPs (endophenotypes, biomarkers) link gene-environment effects on risk behaviors (unhealthy eating habits and sedentary life styles) to detrimental health outcomes (obesity, diabetes, osteoporosis).

Intermediate phenotypes (IPs) are traits or outcome measures that mediate the effects of gene-environment influences on risk behaviors [23]. Such measures tend to be more proximal to the biological determinants than the risk behaviors themselves, and therefore, they can be assessed with greater experimental control in human models. The inclusion of measurable intermediate phenotypes will better assist investigators in the exploration of the relationship among gene-environment interactions, risk behaviors, and health, as shown in Figure 1 [24].

The terms GxE and risk behavior in the boxes from Figure 1 are not two independent factors. The flowchart from the basic predictive model assumes that the environment, by interacting with genes, influences individual factors and that both result in risk behaviors. Such behaviors are then assumed to affect body composition, which leads to deleterious health outcomes such as OB, T2D, and OSTP. Nevertheless, the key component of this workflow is to highlight the site of action from the intermediate phenotypes linking GxE effects with risk behaviors. 

The terms intermediate phenotype, endophenotype, and biomarker have often been used interchangeably in the literature, yielding conceptual confusion. The hypothetical relations among biomarker, intermediate phenotype, and endophenotype reveal that all endophenotypes and intermediate phenotypes are subsets within a greater domain of biomarkers. However, not all biomarkers are necessarily either intermediate phenotypes or endophenotypes. Only a subset of intermediate phenotypes can be regarded as endophenotypes [24].

Nevertheless, some definitions can be outlined. An endophenotype is a measurable component, unseen by the unaided naked eye that lies along or within the pathway between disease (observable phenotype) and distal genotype. Thus, it is internal and not easily discerned without some technological assistance with appropriate sensitivity [25]. Biomarker is a term that is used to designate a molecular or biological marker. In this paper, we use this concept as a metabolic biomarker due to the diseases presented in the model (OB, T2D, OSTP). The most important criteria for a biological marker to be considered as such include that it is associated with a pathological process in a given population, it is inherited, and that it tends to come from linked and inherited genes [26]. 

**Figure 2:**
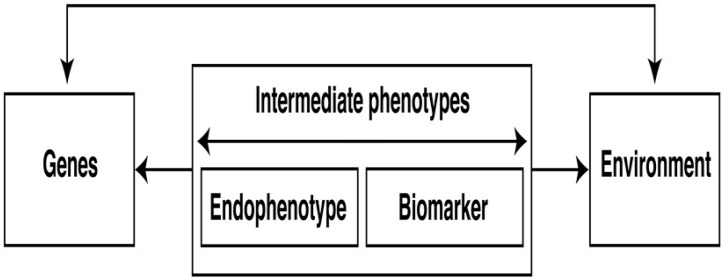
Endophenotypes and biomarkers should be considered subsets of intermediate phenotypes (IPs).

In this paper, we suggest that endophenotypes and biomarkers should be considered subsets within a greater domain of intermediate phenotypes, within the context of a GxE interaction, in order to mediate risk behaviors and influence health outcomes (Figure 2). Independently of the precise conceptual meaning, in this paper, the three terms (intermediate phenotype, endophenotype, biomarker) are used to denote a true link between genes, the environment, risk behaviors, and health outcomes [27].

## 5. Models and Mechanisms of Gene, Environment, and Behavior Interactions in Disease

**Figure 3:**
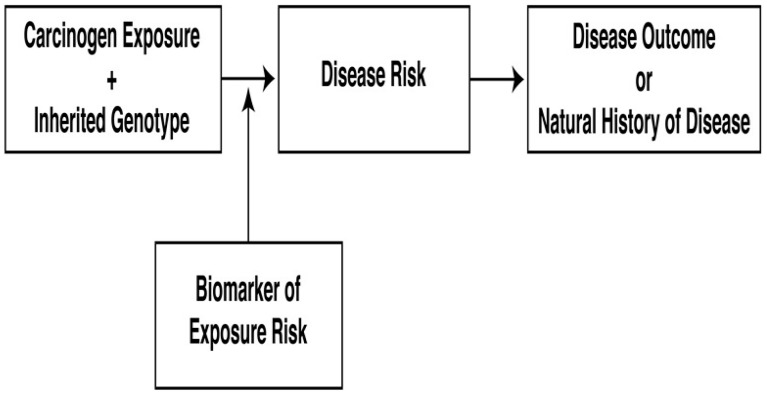
Rebbeck’s predictive multistage model of carcinogenesis [29].

There are many ways to conceptualize the ultimate effect of gene-environment interactions on the expression of a trait or behavior. For example, some genetic effects may be seen only under certain environmental conditions, or some environmental effects may be seen only under certain genetic conditions. In addition, some genetic effects may influence the environment to which an individual is exposed, or genetic and environmental factors may contribute independently to the outcome [28]. A good example is the predictive multistage model of carcinogenesis which describes the progression of normal cells to initiate their abnormal progression to preneoplastic cells, and finally to malignant and metastatic disease (Figure 3). Rebbeck presented this predictive model showing how a number of classes of genes acting in multistage carcinogenesis may explain their contribution to breast cancer. The author concluded that understanding the role of inherited genotypes at different stages of carcinogenesis could improve our understanding of breast cancer biology, may identify specific exposures or events that correlate with carcinogenesis, or target relevant biochemical pathways for the development of preventive or therapeutic interventions [29].

**Figure 4:**
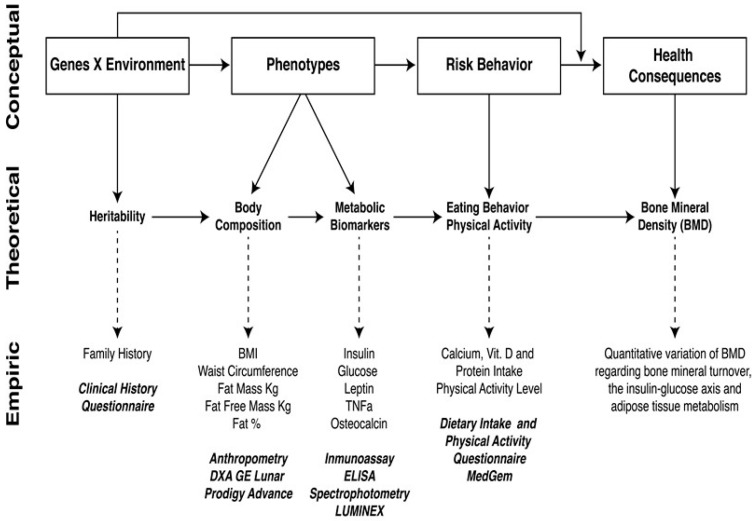
Predictive model of the conceptual-theoretical-empirical GxE influences on BMD and susceptibility to osteoporosis risk within the context of the insulin-glucose axis, and adipose tissue metabolism [31,32].

The predictive model of carcinogenesis can be adapted to common, complex, highly prevalent metabolic diseases such as T2D, OB, and OSTP (Figure 1). This model can be not only modified but amplified in the search for early biomarkers or intermediate phenotypes. As part of a bi-national, multi-center collaborative study of cardiovascular risk phenotypes of a metabolic origin related to T2D and the risk of cardiovascular disease called the GEMM (Genetics of Metabolic Diseases in Mexico) Family study [30], Nava-Gonzalez and Gallegos-Cabriales published a research protocol with the aim of determining the normal quantitative variability of bone mineral density (BMD) in healthy adult females. They associated it with phenotypes determined by measurements of body composition and circulating metabolic biomarkers corresponding to bone physiology (mineral bone turnover determined by osteocalcin) and the activity of the insulin-glucose axis and adipose tissue, in order to establish deleterious consequences to health such as OSTP, T2D, and OB [31]. The authors utilized a research design model based on the Rebbeck proposal for breast cancer [29] (Figure 3) by amplifying and adapting the cancer model to an innovative predictive model to study the disorders of body composition (Figure 4). The results of this paper presented the relevance of a predictive model utilizing intermediate phenotypes of body composition and adipose tissue metabolism, including metabolic biomarkers related to the insulin-glucose axis, allowing correlations with BMD variation in order to establish the biological, clinical, and epidemiological relationships between bone and adipose tissue [32].

**Figure 5:**
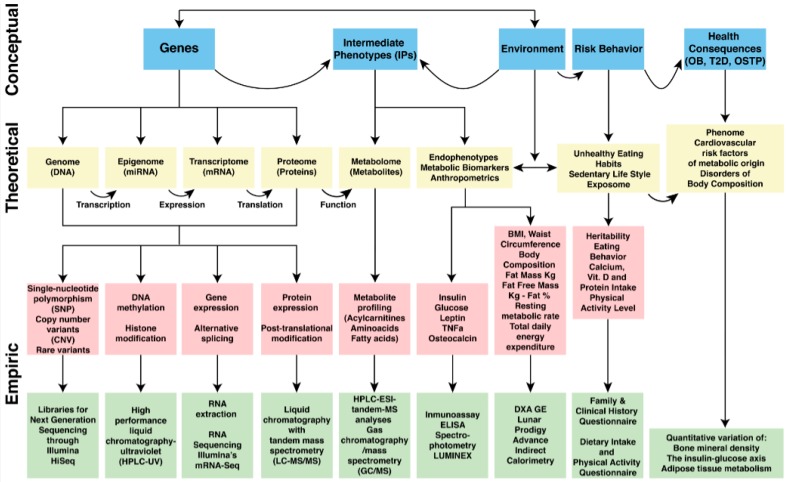
Nava/Gallegos predictive model of GxE interactions in the context of the disorders of body composition with the hypothetical inclusion of data to integrate intermediate phenotypes with biological systems multi-OMICS.

In theory, the authors believe that the model is expandable and able to be modified according to research needs and the aim of a study design regarding common, complex disorders of body composition. It can also be adapted to the newest endophenotypes of an OMICS origin if the research design is planned to include such measurements [33]. This seems to be an appropriate future approach to design studies including OMICS endophenotypes (metabolomics, proteomics, transcriptomics) and intermediate (biomarkers and clinical) phenotypes in predictive models for common metabolic diseases of body composition (Figure 5) [34]. 

## 6. Beyond Health Consequences: Deep Phenotyping for Early Biological Prevention

Observable traits above the molecular level (phenotypes or the phenome as it is currently termed) are currently driving much of the research in life sciences. In contrast to phenotypes, annotation strategies for the OMICS (genome, proteome, transcriptome, metabolome) are well advanced, with common methodologies, tools, syntaxes, and standards for articulating a precise description of nearly every type of genomic element. In spite of these scientific advancements, much of the genetic etiology of complex traits and bio­logical networks remains undiscovered. Large-scale exome and whole-genome sequencing studies that are focused on protein-coding regions of the genome have accounted for only about 40% of the genetic basis of common, complex, highly prevalent traits. This dilemma is termed the “missing heritability” and has been puzzling geneticists for more than a decade [35]. Using the genetic basis of obesity as an example, an immense number of genetic variants for obesity susceptibility have been found. As stated, these variants explain only a very scant part of the genetic contribution to the development of the disease [36]. In recent years, a different critique has arisen indicating that perhaps most genetic effects upon body weight are likely to become obscured by the use of inappropriate phenotypes. In particular, clinical categories such as the body mass index (BMI) do not provide sufficient etiological information to be used sensibly in genetic studies on obesity or obesity-related disease [37]. Therefore, the scientific community argues in favor of much better and deeper phenotypes [38]. 

Deep phenotyping is defined as the comprehensive analysis of phenotypic abnormalities in which the individual components of the phenotype are observed and described, often for the purposes of scientific examination of human disease. There are examples that might show the research advantages of employing the deep-phenotyping approach: (a) Data obtained from a longitudinal study design making multiple measurements over time with fairly short time periods between measurements, would enable the investigator to detect dynamic changes in the nature of the phenotype; (b) A mean biomarker value calculated based on two or three blood draws spread over the day is likely to eliminate within-subject variability. Therefore, the case of deep phenotyping is likely to reduce the inaccuracy and misclassification of disease outcomes present in most epidemiological and clinical studies, by increasing an individual’s phenotypic information and refining risk classification [39]. Deep phenotyping seems to accurately fit for measurements of postprandial metabolism. Recent trends in genetic and molecular research study designs for metabolic diseases utilize dynamic rather than static phenotypes to measure the differential response of the fasting and fed state. Their focus is the functional aspects of the disease process [40]. The function-based deep phenotype approach will allow us to identify inter-individual differences in metabolic adaptation to weight changes in future genetic studies on obesity and other metabolic diseases in order to achieve early biological prevention strategies [41].

## 7. Beyond Risk Behaviors: The Exposome 

The main environmental factors contributing to the development of OB and T2D include a lack of physical activity; a sedentary life style; unhealthy eating habits; highly palatable, inexpensive food availability; and large portion sizes. In recent years, epidemiologists have concluded that a more comprehensive and quantitative view of environmental exposure and risk behavior is needed in order to discover the major causes of chronic complex diseases. There is a need to develop methods with the same precision for an individual’s environmental exposure as we have for the individual’s genome. The term exposome has been coined to match the genome [42]. The exposome considers the environment as the body’s internal chemical environment, and exposures as the amounts of biologically active chemicals in this internal environment. Such exposures are not restricted to chemicals entering the body from air, water, or food, for example, but also include chemicals produced by inflammation, oxidative stress, lipid peroxidation, infections, gut flora, and other natural processes. The characterization would comprise a profile of the most prominent classes of endocrine disruptors, modulators of immune responses, agents that bind to cellular receptors, and metals [43]. Exposures to these agents can be monitored in the blood either by direct measurements or by looking for their effects on physiological processes such as metabolism. These processes generate products that serve as signatures and biomarkers in the blood. It is pertinent to ask whether the new OMICS technologies of transcriptomics, proteomics, and metabolomics can help unlock the problem of environmental exposure assessment. Currently, these methods are mainly applied to the understanding of disease mechanisms and diagnosis. Therefore, an extension of the current generation of biomarkers, together with an evaluation of the new generation of OMICS technologies applied to characterize the exposome, may have a crucial role to play in the near future [44].

## 8. Beyond Intermediate Phenotypes: Systems Genomics Methods

Despite decades of intense research, a large part of molecular pathogenesis and the genomic basis of highly prevalent cardiovascular risk phenotypes of a metabolic origin (excessive accumulation of body fat, adiposopathy, dysglycemia, insulin resistance, hyperinsulinemia, and dyslipidemia) remain unexplained. Our ability to identify the genetic variation that underlies these complex traits and their biological networks could be limited by routinely focusing our efforts on restrictive single-data-type study designs utilizing analytical strategies such as linkage analysis in family-based data, RNA sequencing (RNA-seq), methylation arrays, metabolomic, or proteomic studies to independently identify DNA sequences, epigenetics, protein variation, or gene expression [45].

Researchers are currently utilizing meta-dimensional analysis and multi-staged analysis (that is, systems genomics approaches) to achieve a more thorough and informative interrogation of genotype–phenotype associations than an analysis that uses only a single data type. They are combining multiple data types such as gene expression using microarrays and RNA sequencing (RNA-seq), and protein variation (assayed in either metabolomic or proteomic studies) to interrogate the complete biological model and obtain results on the different levels of genetic, genomic, transcriptomic, metabolomic, and proteomic regulation. Biological systems multi-omics can potentially be characterized at several levels: at the genomic level through single-nucleotide polymorphism (SNP), copy number variation (CNV); at the epigenome level through DNA methylation, histone modification, and micro RNA (miRNA); gene expression and alternative splicing at the transcriptome level; protein expression and post-translational modification at the proteome level; and metabolite profiling at the metabolome level, and, ultimately, at the phenome and exposome level [46].

## 9. Conclusions

GxE interactions can be explored by using predictive models which take into account the various ways in which genetic effects can be modified by environmental exposures. The subject of genetic and environmental influences on the disorders of body composition (OB, T2D, and OSTP), and how they interact, is a unique topic for which conceptual frameworks are scarce. Our paper addresses predictive models to design research studies on three highly complex, multifactorial, polygenically determined, developmental, and environmentally-dependent diseases of major importance to today’s research, science and society: obesity [47], Type 2 diabetes [48], and osteoporosis [49]. The models in Figure 4 and Figure 5 attempt to integrate a research strategy to simultaneously utilize IPs and correlate them better with measurements of the environment, its risk behaviors, and the outcomes represented by these three conditions as a whole with accurate measurements of the genome, in order to target an accurate and deeper research direction to unravel ultimate causality or a common soil for these pathologies [50]. In particular, the purpose of Figure 5, although somewhat complex, is to serve as a guidance for study design planning on measurements from the environment and/or the genome if research work is being pursued on disorders of body composition. 

These frameworks, translated into predictive models to develop systems biology research designs, seem to offer an adequate strategy. Such predictive models should integrate large-scale genetic, intermediate (molecular) phenotypes (IPs), and disease (clinical) phenotypes. Traditional genetic studies identify genetic loci and allelic variants associated with clinical disease phenotypes which provide causal information but lack mechanistic insights. Molecular profiling experiments help identify IPs correlated with disease status, but the results are purely correlative with no causal information. Systems genomics is a new research field that integrates OMICS together with physiological, epidemiological, and environmental data to create a systems network that can be used to predictively model multilevel causes of health and disease. This systems genomics approach is contrasted with the simplicity of the single-level paradigm in classical epidemiology focusing mostly on a single risk factor related to a disease [51]. 

This mini-review offers an overview of the advances in the field of GxE interactions on the disorders of body composition as well as straightforward predictive models and methods used to detect the interactions. It provides up-to-date descriptions of major findings of GxE interactions on OB, T2D, and OSTP. It also highlights the key role of measurable intermediate phenotypes to help understand the relationship among gene-environment interactions, risk behaviors, and health outcomes. With the successful characterization of phenomes, exposomes, and genomes, environmental and genetic determinants of these chronic diseases can be united in high-resolution studies that better examine these GxE interactions.
